# The cost of coping: a cardio-neuro-metabolic risk for black South Africans?

**Published:** 2010-08

**Authors:** Leoné Malan, NT Malan, A Du Plessis, MP Wissing, JC Potgieter, YK Seedat

**Affiliations:** Hypertension in Africa Research Team (HART), School for Physiology, Nutrition and Consumer Sciences, North-West University, Potchefstroom Campus, Potchefstroom, South Africa; Hypertension in Africa Research Team (HART), School for Physiology, Nutrition and Consumer Sciences, North-West University, Potchefstroom Campus, Potchefstroom, South Africa; Hypertension in Africa Research Team (HART), School for Physiology, Nutrition and Consumer Sciences, North-West University, Potchefstroom Campus, Potchefstroom, South Africa; Psychosocial Behavioural Sciences, North-West University, Potchefstroom Campus, Potchefstroom, South Africa; Psychosocial Behavioural Sciences, North-West University, Potchefstroom Campus, Potchefstroom, South Africa; The Renal Hypertension Unit, Nelson Mandela School of Medicine, University of Kwa-Zulu Natal, Durban, South Africa

Psychosocial stress is a contributing factor to cardiovascular disease.^1^ An important way of investigating the mechanisms underlying this association is acute psycho-physiological stress testing, involving measurement of physiological responses to laboratory-induced stress. Psycho-physiological stress testing allows individual differences in responses to standardised stress to be evaluated and related to psychosocial and cardiovascular risk factors. Accumulating evidence has demonstrated associations of disturbed psycho-physiological responses with subclinical measures of atherosclerosis, hypertension and metabolic risk.^2^-^11^

The complex pattern of responding to stress is influenced by individual differences, such as coping style, race and ethnicity, genetic make-up, background stress, and lifestyle habits, which should be taken into account when interpreting results. For example, a unique interplay between cardiac and vascular responses in urban black Africans^2^ is thought to contribute towards a heightened risk of hypertension in this group. Whether or not psycho-physiological risk markers provide prognostic information over and above that of established risk markers is not clear but adapting to or appraisal of an urban environment could add to heightened responses.

There are, however, marked ethnic differences in cardiovascular responses or reactivity (CVR) that may in part be explained by psychosocial factors. Black Africans^7^,^8^ and African-Americans^4^ normally and when exposed to stressful situations exhibit exaggerated CVR and a higher risk for the development of hypertension^1^ in comparison to Caucasians. In three separate regions, including rural and urban black South Africans, results demonstrated increased vascular responses on exposure to the handgrip and cold pressor tests in urban participants compared to their rural counterparts. It is suggested that in urban Africans the physiological adaptation process is inclined to shift towards increased vascular responses and prevalence of hypertension. We might speculate that the individual who experiences emotional–social–cultural disruption in an urban environment is in a continuous state of hyper-vigilant coping and vascular sympathetic hyperactivity/overdrive through exaggerated CVR.

Mental and chronic psychosocial stress triggers sympathetic nervous system (SNS) hyperactivity and has been shown to be associated with increased vascular responsiveness, pressure overload, hypertension and myocardial ischaemic risk.^5^,^7^ Also, increased vascular and plasma renin responses seen in black African men further increase sympathetic activity through peripheral vascular hyper-responsiveness and higher blood pressure.^6^,^7^,^9^ This change to a high vascular resistance pattern accompanying hypertension predisposes to the development of left ventricular hypertrophy. In fact, studies show that African-Americans have greater left ventricular wall thickness than Caucasians, suggesting that increased peripheral vascular resistance may be due to structural changes in the vasculature. However, it is not certain whether this is also true for black Africans.

The concept of coping style or strategies implies that humans have a differential capacity to adapt to the same environmental conditions. When attended to and appraised in certain ways, a coordinated set of responses involving behavioural and physiological systems is triggered.^3^ Mainly, two different appraisals or stress coping responses may be distinguished: firstly, defensive problem-solving active coping (high AC), seeking social support and being-in-control responses, which elicit fight-or-flight b-adrenergic central cardiac responses. Secondly, avoidance coping (low AC) and loss-of-control responses which elicit defeat α-adrenergic vascular responses and are associated with pathology.^3^ If a coping strategy fails, stress pathology may develop because of an over-arousal of the sympathetic nervous system, thereby increasing the risk for related cardiovascular diseases (CVD).^4^

It is difficult to fully elucidate if an enhanced stress response is dependent on the appraisal of the stressor, and if mental stress evokes the same stress response pattern in groups of different ethnicity. What is however evident from animal studies is that stress and re-exposure illicit different mono-aminergic (nor-adrenaline, dopamine and serotonin) responses in cortical and sub-cortical (e.g. hippocampus) brain regions, resulting in behavioural changes, and which have their origin in altered functioning of the hypothalamic–pituitary–adrenal stress axis. Appraisal or detection of stressful stimuli at conscious and subconscious levels involves the thalamus, sensory and prefrontal cortex. Reciprocal connections exist with several sub-cortical pathways, determining autonomic and emotional responses through the hypothalamus and amygdala.^5^ Defensive high active coping responses are generated in a reward centre, i.e. the paraventricular nuclei of the hypothalamus, secreting vasopressin, involving sub-cortical pathways via central cardiac β-adrenergic response patterns.^5^

Animal studies indicate that while acute stress significantly elevates nor-adrenalin levels, especially in the hippocampus, a significant attenuation is experienced following re-experience. Interestingly, this does not occur in the frontal cortex, suggesting that subsequent behavioural changes following re-stress are driven by adrenergic-mediated changes in the hippocampus, such as deficits in cognition. The latter will compromise stress coping and stress responsiveness and in this way ultimately compromise central and peripheral cardiovascular response mechanisms. Hence a sensitisation of defensive pathways may occur, with α-adrenergic vascular pathways gaining influence. Consequently, the risk for established essential hypertension in high-demand situations may increase.^5^

It has also been shown that peripheral vascular adrenergic hyperactivity contributes to human hypertension^8^ and is characterised by (1) down-regulation of α-adrenergic receptors, (2) an impairment in the neuronal uptake of nor-epinephrine from sympathetic nerve terminals, and (3) an altered functional interaction at the level of the vascular wall between nor-epinephrine, epinephrine, and other humoral (such as angiotensin II), metabolic (including insulin and leptin), or endothelium-derived substances, e.g. asymmetrical dimethylarginine (ADMA).^8^ Altogether, the above proposed mechanisms coupled to lowrenin hypertension,^6^ salt sensitivity^1^ and increased vascular responses in Africans^2^,^6^,^7^,^9^ and African-Americans may contribute to an increase in peripheral vasoconstrictor tone, as well as an abnormal vasomotor responsiveness to adrenergic stimuli in hypertension.

Overall, subjects maintain their characteristic response tendencies (central or vascular) across mental tasks but it was also shown that when the task was long enough, certain individuals shifted from cardiac to vascular responders. This could support the findings of Malan *et al.*^7^,^9^ where α-adrenergic responses on exposure to acute mental stress were elicited in high AC Africans in a rural environment opposed to α-adrenergic responses in high AC Africans in an urban environment. Therefore, we might propose that if a hyperkinetic–hyperactive peripheral adrenergic sympathetic drive is elicited during acute/chronic stress, coupled to dissociation between a behavioural and a physiological defensive high AC α-adrenergic coping style, it could imply uncontrollable stress.^2^ A resultant high vascular resistance pattern emerges, augmenting possible inward eutrophic vascular remodelling and inflammation, a hallmark of hypertension.^5^

**Fig. 1.**
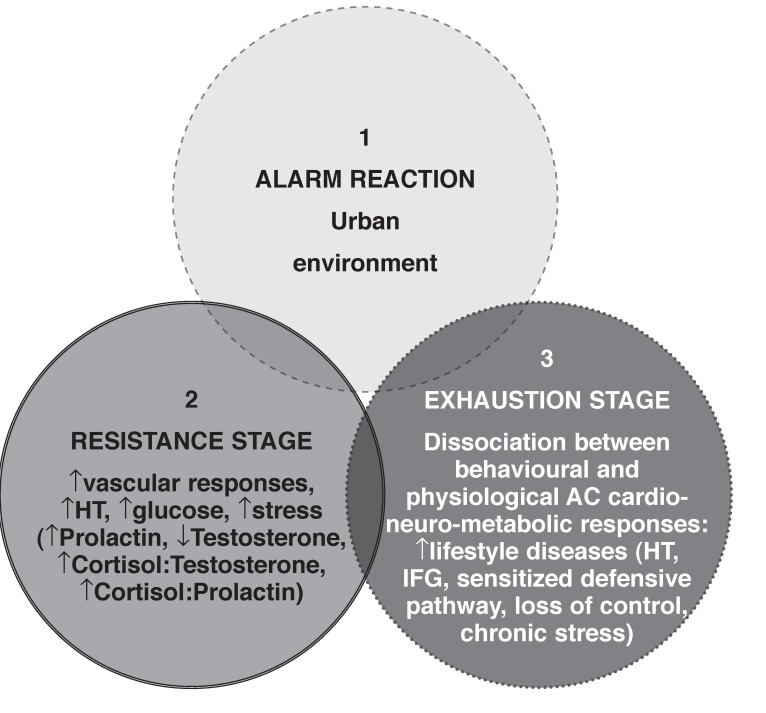
Implementing the general adaptation syndrome^10^ to interpret the dissociation between behavioural and physiological AC cardio-neuro-metabolic responses in urban black Africans. HT = hypertension; AC = active coping; IFG = impaired fasting glucose.

Implementing Fig. 1, these data suggest a physiological adaptation process of black Africans associated with an urban environment or chronic psychosocial stress. Moving from a traditional rural African setting (a collectivistic cultural context with support networks) to an urban area (an individualistic cultural environment without social support) is likely to exacerbate stress. Over time, the combined effects of stress, enhanced vascular reactivity, associated with α-adrenergic stimulation and synergistic effects on cortisol may further impact on depression/stress via the hypothalamus–pituitary–adrenal cortex axis (submitted to *Biol Psych* 2010) and predispose these individuals to greater cardiovascular risk. Clearly, our understanding of these interactions is critical for the development of recommendations for early prevention of hypertension and the metabolic syndrome in urbanised Africans.

Future studies are, however, required to establish if stress responses to behaviourally induced stressors could prospectively predict hard clinical CVD endpoints, such as myocardial infarction and cardiovascular death. Laboratory-induced responses are relatively stable within an individual and consistent across time, although the responses might not always be reflective of everyday life. Therefore, future work is required that examines not only a battery of laboratory measures but also the associations of ambulatory responses to real-life stress in relation to risk of CVD.

The first well-controlled psycho-physiological study in Africa, the SABPA I study (Sympathetic activity and Ambulatory Blood Pressure study on Africans) was conducted in 2008/2009. It is an example of such a study where genetic polymorphisms, adrenergic, pro-inflammatory, prothrombic, blood pressure, heart rate variability (HRV), ischaemic events, oxidative stress, and stress hormone responses to acute mental stress were evaluated in urban teachers from South Africa. In addition, the study will incorporate ambulatory measures during the normal working day. Preliminary findings from SABPA I indicated that the prevalence of HIV/AIDS in the black SABPA teachers was only 9%, opposed to the overwhelmingly increased hypertension prevalence rates in the black teachers [males (79%), females (45%)]. This implies that CVD risk attributable to other risk factors apart from the inflammatory status contributed to their overall health profile, and risk factors should be broadened without concentrating on only the HIV/AIDS factor. Data from the SABPA I and SABPA II follow-up (2011/2012) studies will therefore provide an estimate of the health/disease burden attributable to established and emerging risk factors for CVD, metabolic syndrome, psychological distress and progression of subclinical atherosclerosis.

Future studies should examine whether non-pharmacological interventions that reduce SNS hyper-responsiveness, such as physical activity and/or active relaxation breathing techniques could lessen sympathetic drive and increase vagal outflow.
